# Berberine Delays Onset of Collagen-Induced Arthritis through T Cell Suppression

**DOI:** 10.3390/ijms22073522

**Published:** 2021-03-29

**Authors:** Alexandra A. Vita, Hend Aljobaily, David O. Lyons, Nicholas A. Pullen

**Affiliations:** 1 School of Biological Sciences, University of Northern Colorado, Greeley, CO 80639, USA; alexandra.vita@unco.edu (A.A.V.); david.lyons@unco.edu (D.O.L.); 2 Department of Applied Statistics and Research Methods, University of Northern Colorado, Greeley, CO 80639, USA; aljo5297@bears.unco.edu

**Keywords:** arthritis, berberine, RA, autoimmune, T cell, T_fh_ cell

## Abstract

There is evidence that berberine (BBR), a clinically relevant plant compound, ameliorates clinically apparent collagen-induced arthritis (CIA) in vivo. However, to date, there are no studies involving the use of BBR which explore its prophylactic potential in this model of rheumatoid arthritis (RA). The aim of this study was to determine if prophylactic BBR use during the preclinical phase of collagen-induced arthritis would delay arthritic symptom onset, and to characterize the cellular mechanism underlying such an effect. DBA/1J mice were injected with an emulsion of bovine type II collagen (CII) and complete Freund’s adjuvant (day 0) and a booster injection of CII in incomplete Freund’s adjuvant (day 18) to induce arthritis. Mice were then given i.p. injections of 1 mg/kg/day of BBR or PBS (vehicle with 0.01% DMSO) from days 0 to 28, were left untreated (CIA control), or were in a non-arthritic control group (*n* = 15 per group). Incidence of arthritis in BBR-treated mice was 50%, compared to 90% in both the CIA and PBS controls. Populations of B and T cells from the spleens and draining lymph nodes of mice were examined on day 14 (*n* = 5 per group) and day 28 (*n* = 10 per group). BBR-treated mice had significantly reduced populations of CD4^+^T_h_ and CD4^+^CXCR5^+^ T_fh_ cells, and an increased proportion of Foxp3^+^ T_reg_ at days 14 and 28, as well as reduced expression of co-stimulatory molecules CD28 and CD154 at both endpoints. The effect seen on T cell populations and co-stimulatory molecule expression in BBR-treated mice was not mirrored in CD19^+^ B cells. Additionally, BBR-treated mice experienced reduced anti-CII IgG2a and anti-CII total IgG serum concentrations. These results indicate a potential role for BBR as a prophylactic supplement for RA, and that its effect may be mediated specifically through T cell suppression. However, the cellular effector involved raises concern for BBR prophylactic use in the context of vaccine efficacy and other primary adaptive immune responses.

## 1. Introduction

Rheumatoid arthritis (RA) is a systemic autoimmune disease typically characterized by chronic inflammation and deterioration within the joints. Extra-articular and systemic manifestations can also be present depending on the severity of the disease, and some individuals may experience damage to organs such as the heart, lungs, kidneys, and skin [1]. To date, there are a number of well-described treatments available for clinically apparent RA. Of these treatments, conventional disease-modifying antirheumatic drugs (DMARDs) and biological DMARDs, also known as biologics, are the most effective for long-term management of RA. However, the effectiveness of these treatments at managing disease progression varies among patients [1], and can be influenced by genetic factors [2,3] and the duration of symptoms prior to the first treatment [4,5,6]. Such interpatient variability in terms of response to medication can interfere with a patient’s ability to achieve remission and/or the desired level of disease activity, and can also interfere with a patient’s ability to adhere to a treatment regimen due to reasons of toxicity, lack of efficacy, and/or high cost [7,8,9,10].

Due to the large physiological and economic burden this disease places on its patients, research has become increasingly focused on ways to identify and develop effective preventative treatments targeting RA during the pre-clinical phase of the disease and thereby delay the onset of clinical RA [6,11,12,13]. The pre-clinical phase is commonly defined as the stage of the disease in which an individual experiences local or systemic autoimmunity, evidenced by serological abnormalities (e.g., high levels of CRP, TNF-α, etc.) and/or autoantibodies (e.g., anti-cyclic citrullinated peptide (ACPA), rheumatoid factor (RF), etc.) in the absence of clinical arthritis [13,14]. Targeting individuals in the pre-clinical phase of the disease with preventative therapies would provide the earliest initiation of treatment possible and could halt disease progression prior to significant joint damage. Furthermore, since the inflammatory load is far less in patients in the pre-clinical phase than in patients experiencing clinical arthritis, it presents an opportunity to potentially use lower-cost, broader spectrum complementary therapies that may be less affected by interpatient variability than conventional DMARDs and biologics, which act through specific, targeted pathways.

Berberine (BBR) is a plant-derived isoquinoline alkaloid found in the roots, rhizomes, and stem bark of plants among a variety of genera, such as *Berberis* (its namesake)*, Mahonia* , * Hydrastis*, and *Coptis*, among others. BBR merits further exploration as a potential prophylactic therapy as it has already proved to be of importance for a variety of diseases through successful clinical trials [15,16,17,18,19,20,21]. As such, much is already known about the general toxicology and common side effects of BBR in humans, which are considered to be mild (e.g., diarrhea, flatulence, abdominal pain, and nausea), and do not occur in all patients [15,17,18,22]; there were no adverse effects observed on liver and kidney function [17,18,23]. Notably, amelioration of side effects in patients was reported once the dosage was lowered [18]. 

As an anti-inflammatory, BBR successfully suppresses the inflammatory responses involved in clinically apparent autoimmune diseases in vivo such as collagen-induced arthritis (CIA; a rodent model of RA) [24,25,26,27], type I diabetes mellitus [28], ulcerative colitis (UC) [29,30], and experimental autoimmune encephalomyelitis (EAE) [31]. In regard to RA specifically, BBR has been successful at treating clinically apparent CIA and other RA animal models in vivo through a number of suggested mechanisms, such as (**a**) dendritic cell apoptosis [24], (**b**) interference with MAPK signaling via inhibition of p-ERK, p-38, and p-JNK [25,32], (**c**) attenuation of T_h_17 activity via inducing cortistatin in the gut [27], (**d**) restoration of the balance between T_reg_/T_h_17 cells [26], (**e**) the suppression of T_h_17 differentiation/proliferation through inhibition of CD169 and the RORγt transcription factor, (**f**) induction of T_reg_ differentiation through aryl hydrocarbon receptor (AhR) activation [33], (**g**) and promotion of anti-inflammatory M2 macrophage polarization through upregulation of p-AMPK and inhibition of HIF1α [34]. A more detailed account of the anti-inflammatory actions of BBR in the context of rheumatoid arthritis can be found in the recent review by Shen et al. (2020) [35].

Despite evidence that BBR ameliorates clinically apparent CIA, to date there are no studies involving the use of BBR which explore its prophylactic, pre-clinical potential in a CIA mouse model. Thus, we examined such effects in a CIA mouse model with DBA/1J mice to determine whether or not BBR merits further exploratory analysis as a prophylactic treatment for patients in the pre-clinical phase of RA. The main highlights from this study are:

1. Berberine delays the onset of collagen-induced arthritis in DBA/1J mice.
2. Berberine treatment reduces splenic and lymph node CXCR5^+^ follicular T helper (T_fh_) cell populations.
3. Berberine polarizes splenic and lymph node T cells toward a CD25+ Foxp3+ regulatory (T_reg_) phenotype.

## 2. Results

### 2.1. Berberine Treatment Delays Onset of CIA

**Figure 1:**
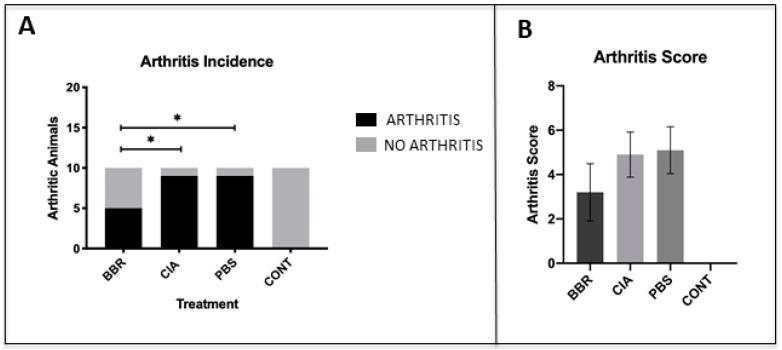
Assessment of collagen-induced arthritis (CIA) in DBA/1J mice in the context of berberine (BBR) treatment. (**A**) Absolute incidence of arthritis (proportions of animals with score ≥2) among treatment groups at day 28 compared using Χ^2^ (*n* = 10 per group, * *p* < 0.05). Incidence proportions were BBR = 50%, CIA = 90%, PBS = 90%, and CONT = 0%. (**B**) Arthritis score, on a scale of 0–16 per manufacturer’s protocol (as described in Materials and Methods), of mice at day 28 treated with BBR (1 mg/kg/day), volume-matched 1X PBS with 0.01% DMSO (PBS vehicle control), or no treatment (CIA control). Multiple comparisons conducted using the Kruskal–Wallis test with Dunn’s multiple comparisons (*n* = 10 per group).

To assess BBR’s ability to delay the onset of clinical CIA, mice were observed daily for signs of redness and joint swelling as an indication of arthritis development, and severity of arthritis was scored on a scale of 0–16 as previously described. When mice were euthanized on day 28, we observed a significant reduction in absolute incidence of arthritis in the BBR group compared to the CIA and PBS controls (Figure 1A). About 90% of mice in both the CIA and PBS control groups developed arthritis, compared to 50% in the BBR group. In mice who developed arthritis, however, there was a trend but no significant difference in severity (Figure 1B).

### 2.2. The Effect of Berberine on Circulating Anti-CII IgG in the CIA Model

**Figure 2:**
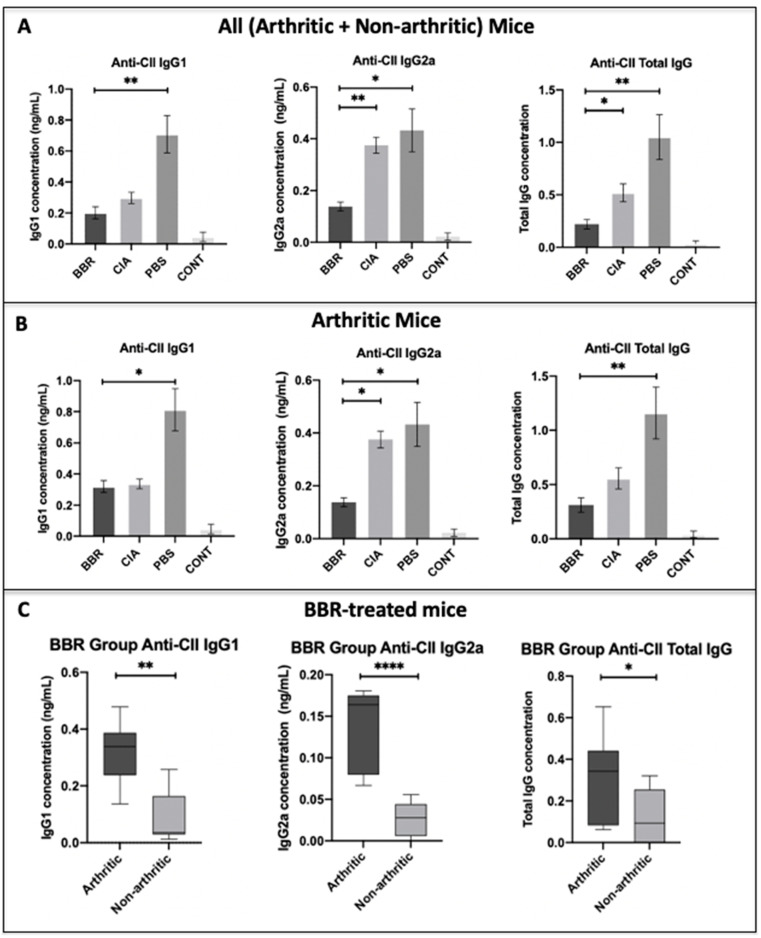
The effect of berberine on circulating anti-bovine type II collagen (CII) IgG in the CIA model. (**A**) Anti-CII IgG1, IgG2a, and total IgG at day 28 among all mice (arthritic and non-arthritic) within BBR, PBS (vehicle control), CIA (no treatment control), and non-CIA control animals (*n* = 10 per group). (**B**) Anti-CII IgG levels at day 28 compared among arthritic mice only (BBR *n* = 5; PBS *n* = 9; CIA *n* = 9). Statistical comparisons made with the Kruskal–Wallis test with Dunn’s multiple comparisons. (**C**) Anti-CII IgG levels at day 28 compared among BBR-treated mice who developed arthritis (“arthritic”) vs. those that did not (“non-arthritic”). Statistical comparisons made with the Mann–Whitney *U* test. For all statistical tests in Figure 2A–C, * *p* < 0.05, ** *p* < 0.01, **** *p* < 0.0001.

To determine if BBR prophylactic treatment reduces autoantibody production, serum concentrations of anti-CII total IgG, anti-CII IgG1, and anti-CII IgG2a autoantibodies were measured at the day 28 endpoint. The BBR group saw significantly reduced serum concentrations of anti-CII IgG2a and anti-CII total compared to both CIA and PBS controls, although there was no significant difference in anti-CII IgG1 in BBR mice compared to CIA control mice (Figure 2A). To further examine if the aforementioned results were an artifact of including both arthritic and non-arthritic mice in the dataset, comparisons of just arthritic mice were performed. In this comparison, levels of anti-CII IgG2a among arthritic mice in the BBR group remained significantly reduced compared to CIA and PBS controls (Figure 2B). When comparing anti-CII IgG levels between arthritic and non-arthritic mice within the BBR group specifically, anti-CI IgG1, IgG2a, and total IgG were all significantly reduced in the non-arthritic mice compared to those who developed arthritis (Figure 2C). Additionally, there appeared to be a vehicle-specific effect on circulating anti-CII IgG in which the administration of PBS with 0.01% DMSO elicited elevated levels of anti-CII IgG1 and total IgG in vehicle control mice (Figure 2A,B).

### 2.3. Key CD4^+^T Cell Population and Co-Stimulatory Molecule Characteristics in Response to Berberine Treatment

**Figure 3:**
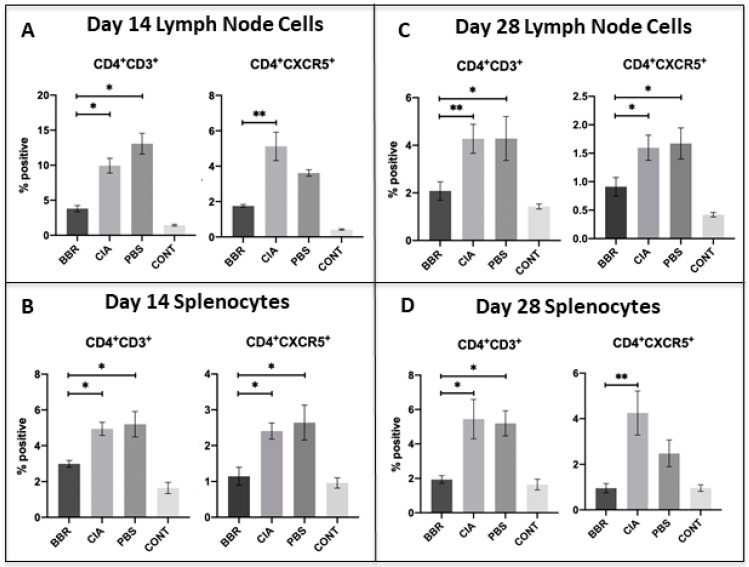
CD4^+^ T cell populations during pre-clinical CIA (day 14) and at final day 28 endpoints. Cells compared were from the CD4^+^ T cell population of lymph nodes (LNs) and spleen with further investigation into CD4^+^ T cell populations expressing specific cell-surface markers. Shown are populations of CD4^+^ T_h_ and CXCR5^+^ T_fh_ cells of the LN (**A**) and spleen (**B**) at the day 14 endpoint (*n* = 5 per group), and of the LN (**C**) and spleen (**D**) at the day 28 experimental endpoint (*n* = 10 per group). Statistical comparisons made with the Kruskal–Wallis test with Dunn’s multiple comparisons (* *p* < 0.05 and ** *p* < 0.01).

**Figure 4:**
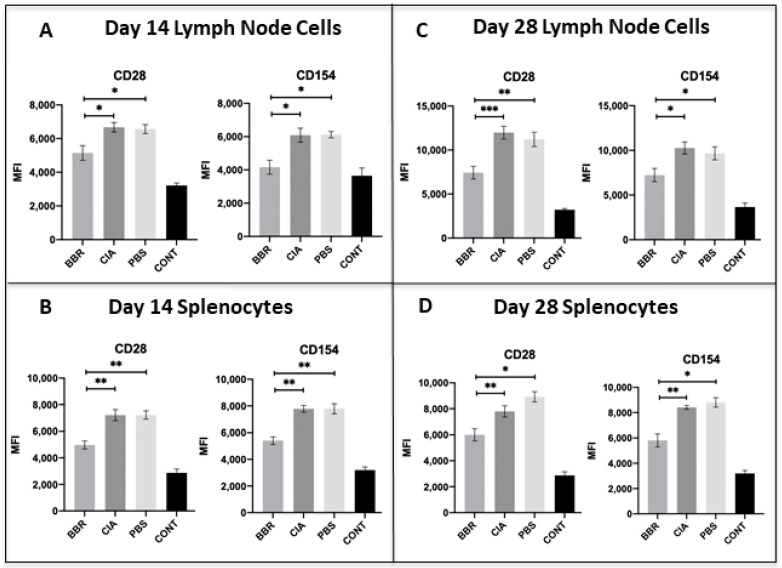
Expression of co-stimulatory molecules on CD4^+^ T cells during pre-clinical CIA (day 14) and at final day 28 endpoints. Cells compared were from the CD4^+^ T cell population of LN and spleen with further investigation into the expression of co-stimulatory molecules CD28 and CD154 on CD4^+^ T cell populations. Shown are expression of CD28 and CD154 on CD4^+^ T cells of the LN (**A**) and spleen (**B**) at the day 14 endpoint (*n* = 5 per group), and of the LN (**C**) and spleen (**D**) at the day 28 experimental endpoint (*n* = 10 per group). Statistical analysis of co-stimulatory molecule expression made with ANOVA and Tukey’s multiple comparisons tests (* *p* < 0.05; ** *p* < 0.01, *** *p* < 0.001).

On day 14, we observed a significant reduction in populations of both CD4^+^T cells and CXCR5^+^T_fh_ cells in the LNs and spleen of BBR-treated mice (Figure 3A,B), as well as a reduction in the expression of CD28 and CD154 on CD4^+^T cells in the spleen and LNs of BBR-treated mice (Figure 4A,B). By the day 28 experimental endpoint, we continued to observe a significant reduction in CD4^+^T cells and CXCR5^+^T_fh_ cells in the spleen and LNs of BBR-treated mice (Figure 3C,D), as well as decreased expression of CD28 and CD154 on the CD4^+^T cells of BBR-treated mice (Figure 4C,D).

### 2.4. Berberine Treatment Leads to Increased Proportion of Foxp3^+^CD4^+^CD25^+^ T Cells

**Figure 5:**
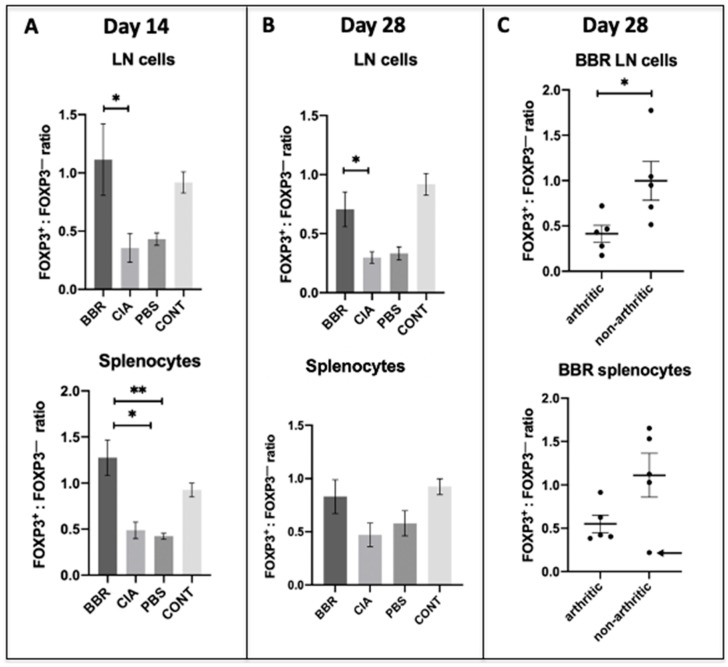
Berberine induces T_reg_ expansion in lymphoid tissue during CIA induction. Cells compared were from the CD4^+^ CD25^+^ T_h_ population of LN or spleen with further interrogation of the definitive T_reg_ transcription factor Foxp3. (**A**) The Foxp3^+^:Foxp3^−^ ratio during the pre-clinical phase of arthritis (day 14) (*n* = 5 per group, * *p* < 0.05). (**B**) The Foxp3^+^:Foxp3^−^ ratio at the day 28 experimental endpoint (*n* = 10 per group, * *p* < 0.05 and ** *p* < 0.01). (**C**) Comparisons of Foxp3^+^:Foxp3^−^ ratios from LN and spleen of the BBR treated group separated by status as arthritic or non-arthritic. Ratios compared using the Kruskal–Wallis test with Dunn’s multiple comparisons. Arrow denotes outlier.

To examine BBR’s effect on T_reg_ populations, cells from the CD4^+^ CD25^+^ T population of LN or spleen were measured for the presence of the definitive T_reg_ transcription factor Foxp3. Out of this subset of cells, we observed an increased ratio of Foxp3^+^:Foxp3^−^ cells in the spleen and LNs of BBR-treated mice during the pre-clinical phase of CIA (day 14 endpoint) (Figure 5A). At the day 28 endpoint, BBR-treated mice had a significantly increased ratio of Foxp3^+^:Foxp3^−^ cells in the LNs, but not the spleen (Figure 5B). In order to determine whether or not the previously mentioned results were an artifact of including both arthritic and non-arthritic mice in the analysis, we compared this ratio between mice in the BBR group who developed arthritis and the mice who did not. There was no significant difference in the day 28 splenic Foxp3^+^:Foxp3^−^ T cell ratio between arthritic and non-arthritic mice in the BBR group. However, all non-arthritic BBR-treated mice had a larger percentage of Foxp3^+^ cells compared to Foxp3^−^ cells (ratio of >1) except for one outlier, whereas all arthritic BBR-treated mice had a smaller percentage of Foxp3^+^ cells compared to Foxp3^−^ cells (ratio of <1) (Figure 5C).

### 2.5. Key CD19^+^B Cell Population and Co-Stimulatory Molecule Characteristics in Response to Berberine Treatment

**Figure 6:**
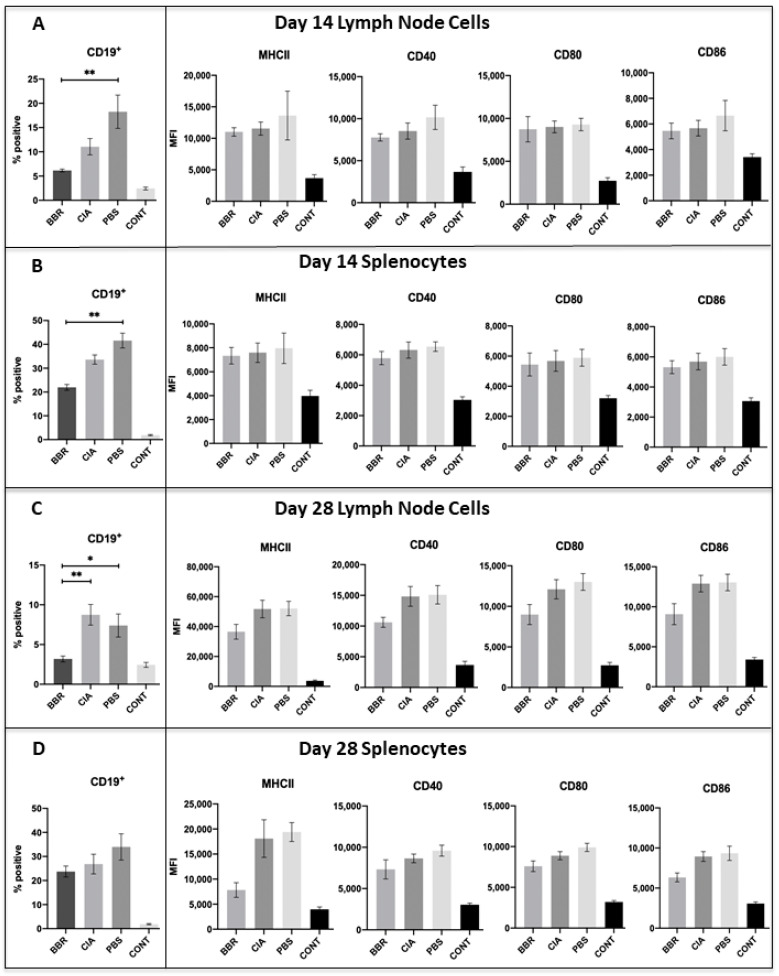
CD19^+^ B cell populations and expression of co-stimulatory molecules during pre-clinical CIA (day 14) and at final day 28 endpoints. Cells compared were from the CD19^+^ B cell population of LN and spleen with further investigation into CD19^+^ cell populations expressing specific cell-surface markers. Shown are populations of CD19^+^ B cells and expression of co-stimulatory molecules MHC Class II, CD40, CD80, and CD86 on CD19^+^ B cells in the LN (**A**) and spleen (**B**) at the day 14 endpoint (*n* = 5), and of the LN (**C**) and spleen (**D**) at the day 28 experimental endpoint (*n* = 10). Statistical analysis of CD19^+^ B cell populations made with the Kruskal–Wallis test with Dunn’s multiple comparisons (* *p* < 0.05), and statistical analysis of co-stimulatory molecule expression made with ANOVA and Tukey’s multiple comparisons tests (* *p* < 0.05 and ** *p* < 0.01).

Although there was a trend of reduced CD19^+^ B cell populations in the spleen and LNs of BBR-treated mice during CIA development (day 14), this trend was non-significant. Additionally, BBR treatment did not reduce expression of the co-stimulatory molecules MHC II, CD40, and CD80/86 on CD19^+^ B cells of the spleen and LNs at day 14 (Figure 6A,B). 

By day 28, we observed a significant reduction in CD19^+^B cells in the LNs, but not spleen, of BBR-treated mice (Figure 6C,D). While there was a trend of reduced expression of the co-stimulatory molecules MHC II, CD40, and CD80/86 on CD19^+^ B cells in the spleen and LNs of BBR-treated mice at day 28, this trend was non-significant (Figure 6C,D). 

We did, however, observe a vehicle-specific effect similar to that seen in the anti-CII IgG data. There were significantly larger CD19^+^ B cell populations in both the spleen and LNs of the PBS control mice compared to the BBR-treated mice, and non-significant trends of increased co-stimulatory molecule expression (Figure 6A,D).

## 3. Discussion

Our results indicate that BBR treatment during the pre-clinical phase of CIA delayed the onset of CIA in DBA/1J mice, although mice in the BBR group who developed arthritis did not experience a significant decrease in clinical arthritis score compared to the CIA and PBS controls. Our results provide evidence at the cellular level that the mechanism underlying this protective effect is directly mediated through effector CD4^+^ T_h_ cell suppression, which subsequently influences activation, proliferation, and autoantibody production by B cells. 

Our hypothesis that BBR is exerting its effect via CD4^+^ T_h_ cell suppression is supported by the observations that BBR-treated mice had significantly reduced populations of CD4^+^ T cells and reduced expression of co-stimulatory molecules—effects which were not mirrored in CD19^+^ B cells. While our CD4^+^ T cell population data included all T cell subsets expressing CD4 (both T_h_ and T_reg_), we observed a higher ratio of Foxp3^+^CD25^+^CD4^+^ T cells (representative of T_reg_) to Foxp3^−^ cells within the spleens and LNs of BBR-treated mice. This indicates that although the overall population of CD4^+^ T cells was smaller in BBR-treated mice, they also had a higher proportion of T_reg_ within the total CD4^+^ T cell population. Additionally, a specific subset of the overall CD4^+^ T cell population which plays a key role in T cell-dependent humoral responses, CD4^+^CXCR5^+^ T_fh_ cells, was also decreased in BBR-treated mice. Together, these observations indicate a preference toward an immunosuppressive environment and specifically a reduced capacity to provide help in activating B cell autoantibody production.

A previous study by Moschovakis et al. (2017) [36] examining the role of CXCR5^+^ T_fh_ cells in RA showed that T cell-specific CXCR5 deficiency prevented RA development. Furthermore, an in vivo CIA study involving the use of hydroxychloroquine (HCQ) as a prophylactic (administered from day 0 of the experiment) noted a reduction in T_fh_ cells, which corresponded to a decrease in both incidence and arthritis score in HCQ-treated mice [37]. Similar to this evidence, it is possible that our observation of reduced populations of CXCR5^+^T_fh_ cells seen in the BBR group compared to the CIA and PBS controls contributed to a lower incidence of arthritis as well. The reduction of CXCR5^+^T_fh_ cells we observed also likely contributed to the decreased generation of anti-CII total IgG and subtypes, as CXCR5^+^T_fh_ cells play a critical role in germinal center formation, B cell affinity maturation, isotype class switching, and subsequent autoantibody production [38]. As such, we propose the mediation of CXCR5^+^T_fh_ cell proliferation as a novel function of BBR, and we are unaware of any studies to date that specifically address the effect of BBR on CXCR5^+^T_fh_ cell populations. 

Additionally, the reduced expression of co-stimulatory molecules CD28 and CD154 during CIA development (day 14) and at the day 28 endpoint in BBR-treated mice could be indicative of reduced activation and proliferation of CD4^+^ T cells, thereby resulting in the lower CD4^+^ T cell populations observed in the BBR-treated group. The blockade to CD4^+^ T cell co-stimulation has proven to be an effective RA treatment and is the mechanism of action of abatacept, a biological immunotherapy used to treat clinically apparent RA [1,39]. CD28-CD80/86 interaction is an important therapeutic target as CD28 ligation leads not only to increased T cell proliferation and activation, but also to increased CD154 expression [40]; CD154 is a crucial ligand involved in the activation of B cells and other APCs, as well as affinity maturation and isotype class switching in B cells. We would also like to highlight that the reduced T cell populations and expression of co-stimulatory molecules was seen throughout the entirety of the experiment (day 14 and day 28). However, since BBR treatment occurred for the duration of the experiment, it is unknown whether the sustained reduction observed is due to the continual administration of BBR, or if ceasing BBR administration would have altered this outcome.

BBR’s protective effect against CIA development is also likely mediated through its alteration of the Foxp3^+^: Foxp3^−^ CD4^+^ T cell ratio. With the exception of the day 28 splenocytes whose data were skewed by one outlier, the BBR group saw a significantly higher proportion of Foxp3^+^CD25^+^CD4^+^ T cells (T_reg_) compared to CIA and PBS controls. Thus, while BBR treatment resulted in lower overall CD4^+^ T cell populations, a higher percentage of cells within that reduced population were T_reg_. Previous studies corroborate the protective effect of T_reg_ on CIA development; adoptive transfer of CD25^+^ T_reg_ slowed disease progression of clinically apparent CIA [41], and the depletion of CD25^+^ T_reg_ prior to immunization with bovine type II collagen (used to induce CIA) exacerbated arthritis [42]. Additionally, prior studies using BBR to ameliorate clinically apparent CIA resulted in a suppression of T_h_17 activity alongside the activation/proliferation of T_reg_, thereby resulting in an increased T_reg_/T_h_17 ratio in BBR-treated mice [26,33]. While a study by Yue et al. (2017) [27] provides opposing evidence in which BBR did not appear to have a significant effect on the frequency of T_reg_ in a CIA model despite seeing amelioration of clinically apparent CIA, their particular model used peripheral blood mononuclear cells to assess the T_reg_ population and Foxp3 expression, as opposed to our study which observed splenocytes and draining LN cells at the site of immune activation. 

In regard to B cell-specific responses to BBR, during CIA development (day 14 endpoint) the BBR-treated mice in our study did not see a significant reduction in overall CD19^+^ B cell populations or expression of co-stimulatory molecules compared to the CIA control. However, by day 28 we observed a significant reduction in CD19^+^ B cell populations in the draining LNs of BBR-treated mice compared to the CIA control, as well as a reduction in anti-CII IgG2a and total IgG. As such, we propose that the reduction in day 28 LN B cell populations and the subsequent lowering of anti-CII autoantibody production are largely due to BBR interfering with the T cell-mediated activation of B cells via T cell suppression, thereby contributing to decreased B cell activation. This interference could be due not only to the decreased CD4^+^CXCR5^+^ T_fh_ cell populations and enhanced proportion of T_reg_ seen throughout the experiment in BBR-treated mice, but also the decrease in the expression of CD28 and CD154 on T cells seen throughout the experiment. Both CD28–CD80/86 and CD154–CD40 interactions play an important role in B cell activation and proliferation, and CD154–CD40 ligation specifically provides key signaling for thymus-dependent humoral immunity responses, such as the isotype class-switching and affinity maturation required to generate high-affinity anti-CII IgG autoantibodies [43,44,45]. Furthermore, previous research has demonstrated that the disruption of CD28–CD80/86 and CD154–CD40 interactions results in reduced anti-CII autoantibody titers, prevention of disease development, and/or amelioration of disease in CIA and other autoimmune arthritis models [46,47,48,49,50]. In other words, pro-inflammatory T cell development and activation is inhibited by BBR early, which leads to a later reduction in B cells reactive to CII stimulus, and this timing fits with classical T cell-mediated B cell activity.

While there was no significant difference in anti-CII IgG1 observed between the BBR group and CIA control, we did observe a significant reduction in anti-CII IgG2a. Moreover, BBR-treated mice who experienced a delay in onset (remained non-arthritic by day 28, despite CIA induction) had significantly lower concentrations of anti-CII IgG1, anti-CII IgG2a, and anti-CII total IgG compared to BBR-treated mice who developed arthritis. In CIA, the IgG subtype that is thought to play the most direct role in inflammation and joint destruction is anti-CII IgG2a, which predominantly activates the complement cascade, although it can also bind Fcγ receptors (FcγR) on FcγR-bearing immune cells. High concentrations of anti-CII IgG1 are also typically present, however, IgG1 more readily binds to and activates FcγR-bearing immune cells and has a lower affinity for activating complement compared to IgG2a [51,52]. The important role of complement activation in CIA pathology is supported by studies that demonstrated amelioration of CIA in response to complement deficiency [53] and that C5-deficient mice were resistant to CIA development [54]. As IgG2a is a strong activator of complement in mice, IgG2a serum concentration has been shown to correlate to the degree of inflammation as well as cartilage and bone destruction in CIA models [55], and reduced serum concentrations of IgG2a were associated with delayed onset and reduced frequency of arthritis incidence [56,57]. However, a notable difference with our study is that while we observed significantly lower concentrations of IgG2a in BBR-treated mice compared to CIA and PBS controls, we did not see any significant difference in the degree of observable inflammation (arthritis scores). Additionally, as previous studies involving the use of BBR to treat clinically apparent CIA reported a significant reduction in anti-CII IgG1 in BBR-treated mice compared to both CIA and PBS controls [24,25], the lack of significant anti-CII IgG1 reduction in the BBR group compared to the CIA control in our own study was unexpected. It is notable, however, that when comparing arthritic and non-arthritic mice within the BBR group alone, the non-arthritic mice had significantly lower concentrations of both anti-CII IgG2a and anti-CII IgG1, indicating that the observed reduced incidence of arthritis is likely in part due to a reduction in circulating autoantibodies, as seen in other studies [56,57]. 

One major unexpected result regarding anti-CII autoantibody production involved a vehicle-specific effect in which the PBS control group saw the highest increase in autoantibody production in comparison to the CIA control and BBR group. Our solution of BBR dissolved in PBS and 0.01% DMSO was modeled in part after a previous CIA study that used BBR dissolved in a PBS/DMSO solution containing a slightly greater concentration DMSO than our own [25]; this previous study did not report elevated levels of anti-CII total IgG or anti-CII IgG subtypes in PBS control groups. However, DMSO has demonstrated the ability to stimulate antibody production in hybridoma cells, which are myeloma-B cell hybrids commonly used to generate large quantities of monoclonal antibodies in research and industry settings [58]. In light of this, it is possible we witnessed a B cell-specific response to the presence of DMSO, and furthermore that treatment of BBR was able to overcome this effect.

In addition to this unexpected vehicle-specific effect, our model also faced limitations. One major limitation was the final day 28 endpoint; prolonging the final endpoint past day 28 would provide more insight into the preventative capabilities of BBR. Due to the fact that our non-arthritic mice continued to have suppressed populations of CD4^+^ T helper cells and CXCR5^+^ T_fh_ cells, higher relative percentages of T_reg_, and lower concentrations of circulating autoantibodies by day 28, we hypothesize that it is likely BBR treatment would at least continue to delay CIA development to a certain point. However, it is not known whether BBR would entirely prevent CIA development in those mice who remained non-arthritic by day 28, or if they would eventually develop symptoms of clinical arthritis at a later timepoint. Additionally, having equal sample sizes between the day 14 and day 28 endpoints would have allowed us to evaluate the progression of the immune response statistically, as opposed to just speculatively. 

This model is further limited in that it assumes a mouse would be able to absorb the i.p. administered dose via oral administration, which is the preferred route of administration for human patients taking BBR dietary supplements. While estimates vary, it is widely known that BBR has an extremely low oral bioavailability (<1%) [59,60,61]. Thus, this model is not entirely reflective of how a human patient would ideally receive BBR as a treatment, nor of how a patient would absorb and distribute BBR as an orally delivered treatment. Finally, this model would have further benefited from a BBR control group, which would have involved receiving a BBR treatment but no CIA induction. This would have allowed us to examine the effects of BBR on the immune system under normal physiological conditions and could have provided us with additional insights into BBR’s immunosuppressive capabilities. 

## 4. Materials and Methods 

### 4.1. General Reagents

DMSO (VWR, Radnor, PA, USA), isoflurane (VetOne, Boise, ID, USA), bovine type II collagen in complete Freund’s adjuvant (Hooke Labs, Lawrence, MA, USA), 1X PBS, berberine hydrochloride (Sigma-Aldrich, St. Louis, MO, USA), ACK lysis buffer (Quality Biological, Gaithersburg, MD, USA), RPMI 1640 supplemented to 2 mM l-glutamine, 1% *v*/*v* penicillin/streptomycin, 1 mM sodium pyruvate, 10 mM HEPES (all from ThermoFisher, Waltham, MA, USA) 0.05 mM β-mercaptoethanol (Bio-Rad, Hercules, CA, USA), and 10% fetal bovine serum (VWR/Seradigm, Radnor, PA, USA).

### 4.2. Berberine Solution

A stock solution of 10 mM berberine dissolved in DMSO was stored at −20 °C when not in use. For all i.p. injections, this stock solution was diluted in PBS for a final DMSO concentration of 0.01% and a BBR concentration of 1 mg/kg when delivered to mice. 

### 4.3. Antibodies

Brilliant Violet 421 anti-mouse CD4 (clone GK1.5), FITC anti-mouse CD3ε (clone 145-2C11), FITC anti-mouse CD19 (clone 1D3/CD19), APC anti-mouse CXCR5 (clone L138D7), Alexa Fluor 647 anti-mouse Foxp3 (clone MF-14), APC anti-mouse I-A/I-E (clone M5/114.15.2), PE anti-mouse I-A/I-E (clone M5/114.15.2), PE anti-mouse CD80 (clone 16-10A1), APC anti-mouse CD80 (clone 16-10A1), APC anti-mouse CD86 (clone GL-1), PE anti-mouse CD40 (clone 3/23), PE anti-mouse CD25 (clone 3C7), APC anti-mouse CD154 (clone MR1), PE anti-mouse CD28 (clone 37.51), and recommended isotype controls (all from BioLegend, San Diego, CA, USA). For interrogating the T_reg_ population, the manufacturer’s protocol was followed for intracellular targets.

### 4.4. Mice

DBA/1J mice (6 weeks old) were purchased from Jackson Laboratories (Bar Harbor, ME, USA). Animals were acclimated to the housing facilities for one week prior to starting experiments and were housed 3 mice per cage in conditions that were in accordance with Institutional Animal Care and Use Committee (IACUC) guidelines to minimize distress. Mice were divided into four groups: Control (no CIA induction, no treatment), CIA (positive control), PBS (CIA induction, given volume-matched vehicle control of PBS with 0.01% DMSO), and BBR (CIA induction, given BBR treatment, 1 mg/kg per day). Before commencing the full experiment involving cellular analyses, a pilot study was performed to determine the efficacy of the CIA model (*n* = 3 per group). Treatments were administered via i.p. injections 5 times per week (5 days on/2 days off) and welfare-related assessments were made daily. In the full study (*n* = 15 per group; 60 animals total), five mice from each group were euthanized on day 14 (pre-clinical stage), with 10 mice from each group being euthanized on day 28. All mice were euthanized via CO_2_ inhalation to effect in accordance with our approved IACUC protocol.

### 4.5. CIA Induction and Assessment

A pre-formulated emulsion of bovine type II collagen and complete Freund’s adjuvant (Hooke Labs, Lawrence, MA, USA) was injected according to the manufacturer’s instructions [62]. Briefly, 0.05 mL of the pre-formulated emulsion were injected subcutaneously near the base of the tail, about 7 to 10 mm distal to the body and at the space in between the ventral and lateral tail veins. This procedure was repeated with all mice in the CIA, CIA + BBR, and CIA + PBS groups, and was considered day 0 of the experiment. For mice undergoing full observation through day 28, on day 18 a booster injection of bovine type II collagen and incomplete Freund’s adjuvant emulsion was given according to the manufacturer’s instructions [62]. On day 28, mice were evaluated for the presence of arthritis, and scored on a scale of 0–16 as follows (per manufacturers’ recommendation [62]): 0 = normal paw, 1 = one or two toes inflamed and swollen, 2 = more than two toes, but not entire paw inflamed and swollen OR mild swelling of entire paw without ankle swelling, 3 = entire paw inflamed and swollen (inclusion of ankle swelling), 4 = severely inflamed and swollen OR ankylosed paw; all paws were assessed for a total possible score of 16. Arthritis scoring was blinded to the researchers. Examples of mouse front and hind paws for each score category can be found in Supplemental Figure S1.

### 4.6. ELISA

Blood samples were collected by cardiac puncture immediately following euthanasia of mice on day 14 and 28. Whole blood samples were then centrifuged to separate plasma from cellular components. Serum concentrations of anti-collagen type II (anti-CII) total IgG (catalog # 1012T), anti-CII IgG1 (catalog # 20321T), and anti-CII IgG2a (catalog # 20322T) were measured by ELISA (Chondrex, Redmond, WA, USA) according to the manufacturer’s instructions [63,64]. Optical densities were taken at 450 nm using a microplate reader. 

### 4.7. Flow Cytometry

Single cell suspensions were made from spleens, inguinal lymph nodes (LNs), and axillary LNs of euthanized mice. Inguinal LNs were chosen in lieu of popliteal LNs because inguinal LNs are equidistant from the site of injection and the site of hind paw inflammation, and so are close enough to both sites to reflect the inflammatory responses of both the injection and the hind paw inflammatory events. To create single cell suspensions, briefly, spleens and LNs were separately ground, washed with 3 mL of ACK lysis buffer for 5 min, and then strained into 35 mL of complete RPMI 1640. Single cell suspensions were then stained with fluorescent antibodies specific for cell lineage markers: CD3^+^CD4^+^Foxp3^−^ T helper (T_h_) cells, CD3^+^CD4^+^CXCR5^+^ T follicular helper (T_fh_) cells, CD3^+^CD4^+^Foxp3^+^ T regulatory cells (T_reg_), and CD19^+^ B cells were measured at day 14 and day 28. Spleen and LN cells were also stained with fluorescent antibodies specific for co-stimulatory molecules involved in T cell and B cell activation and differentiation: CD154 (CD40L) and CD28 on CD3^+^CD4^+^Foxp3^−^ T_h_ cells, CD25 on CD3^+^CD4^+^Foxp3^+^ T_reg_, and MHC class II, CD40, and CD80/86 on CD19^+^ B cells. The expression of these co-stimulatory molecules was measured by calculating the geometric mean fluorescence intensity (MFI) at day 14 and day 28. CD3^+^CD4^+^ staining was used as the parent gate for all the T cell subsets observed in this study. Example gating strategies can be found in Supplemental Figures S2 and S3. 

### 4.8. Statistical Analysis

The assumption of normality was not met for cell population data but was met for co-stimulatory molecule expression data. A chi-square (Χ^2^) test was used to compare absolute arthritic incidence (score ≥ 2) among control and treatment groups. For comparisons of non-normally distributed cell populations, the Kruskal–Wallis test with Dunn’s multiple comparisons or Mann–Whitney *U* test were used. For comparisons of normally distributed co-stimulatory molecule expression data, the ANOVA test with Tukey’s multiple comparisons test was used. All tests had an α = 0.05. All analyses were performed using Prism version 8 (GraphPad, San Diego, CA, USA). 

## 5. Concluding Remarks

In conclusion, BBR likely has protective effects against CIA development by directly suppressing CD4^+^ T helper cell activity, including CXCR5^+^ T_fh_ cells, thus having an indirect effect on B cell activation and autoantibody production. These T cell suppressive effects are evidenced by reduced expression of co-stimulatory molecules on CD4^+^ T cells during CIA development (day 14) and at the final day 28 endpoint, as well as smaller populations of CD4^+^ T cells (including CXCR5^+^ T_fh_ cells), and higher percentages of T_reg_ in BBR-treated mice throughout the experiment. Although populations of CD19^+^ B cells were reduced in the draining lymph nodes of BBR-treated mice by day 28, these suppressive effects are not reflected in other B cell populations throughout the experiment or the expression of co-stimulatory molecules on CD19^+^ B cells of the spleen and LNs, indicating that reduced anti-CII auto-antibody production is likely due to decreased interaction of B cells with activated CXCR5^+^ T_fh_ cells. In the future, it is important to repeat this experiment with a later endpoint to better determine the duration of BBR’s protective effects. Additionally, it is imperative to more closely examine BBR’s influence on CXCR5^+^ T_fh_ cells, as these cells are crucial to the formation of germinal centers, high-affinity class-switched plasma cells, memory B cells, and the humoral immunological memory that is ultimately borne out of germinal center reactions. As such, BBR’s suppressive effect on CXCR5^+^ T_fh_ cell populations, while potentially beneficial for autoimmune pathologies, also raises concern that prolonged use could impact a patient’s ability to mount effective beneficial primary adaptive immune responses. 

## Data Availability

All data generated or analyzed during this study are included in this manuscript.

## Supplementary Materials

The following are available online at https://www.mdpi.com/article/10.3390/ijms22073522/s1.
