# Supplementary material for: Berberine Delays Onset of Collagen-Induced Arthritis through T Cell Suppression
Source: Int J Mol Sci. 2021 Mar 29;22(7):3522. doi: 10.3390/ijms22073522 (PMC8037694; doi:10.3390/ijms22073522)
Supplement: Supplementary file 1 [file ijms-22-03522-s001.zip › ijms-1124904-supplementary.docx]

Score 0

Score 1

Score 2

Score 3

Score 4


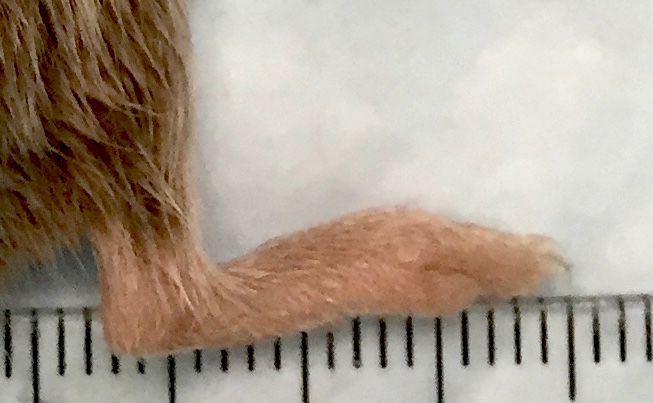

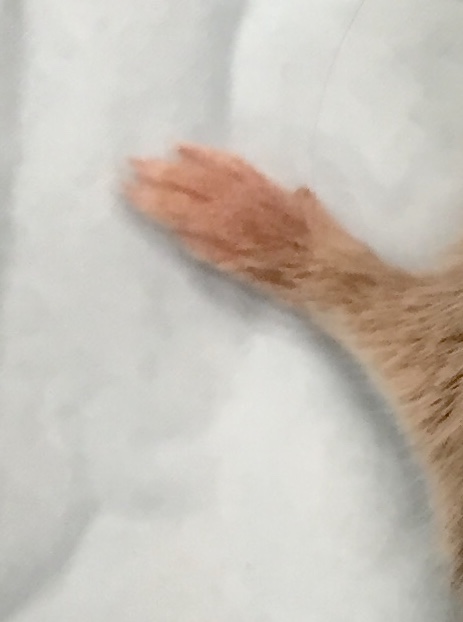

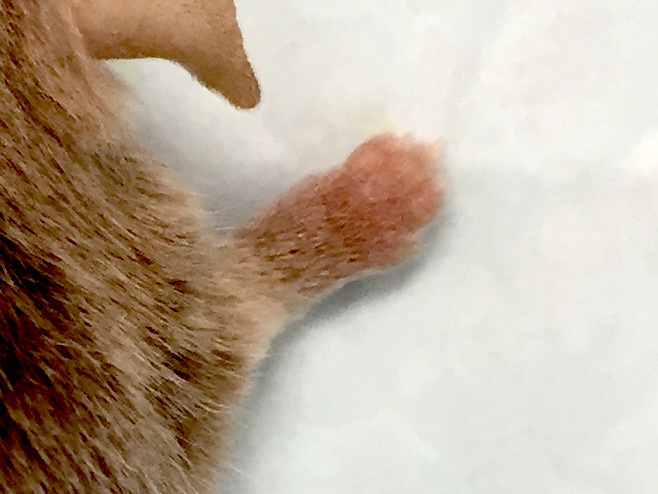

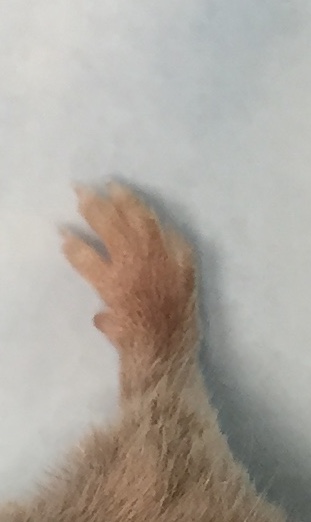

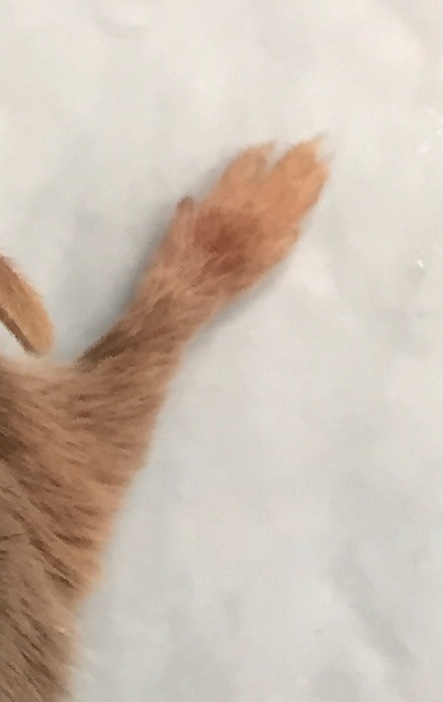

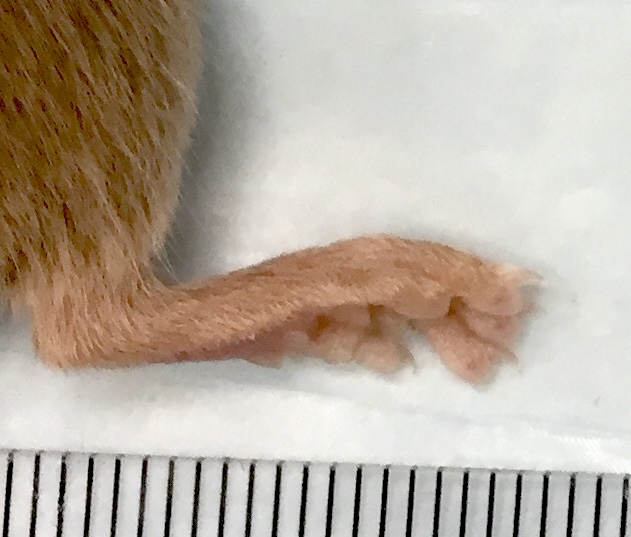

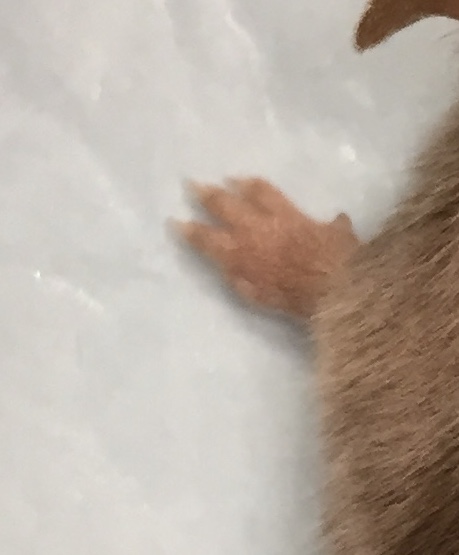

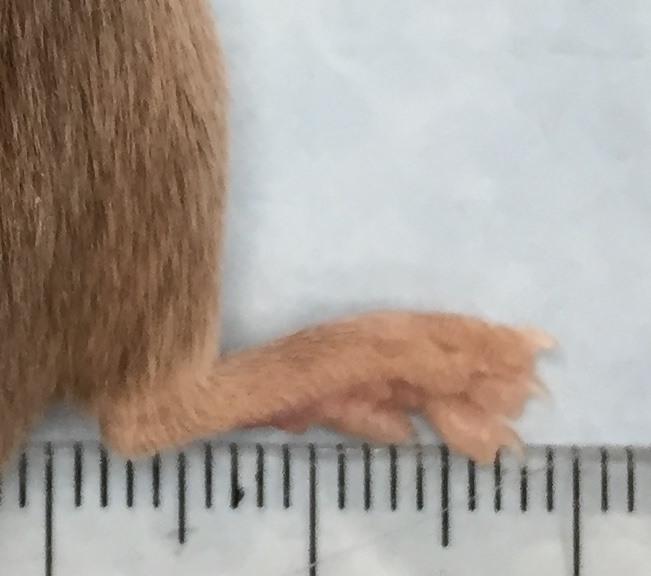

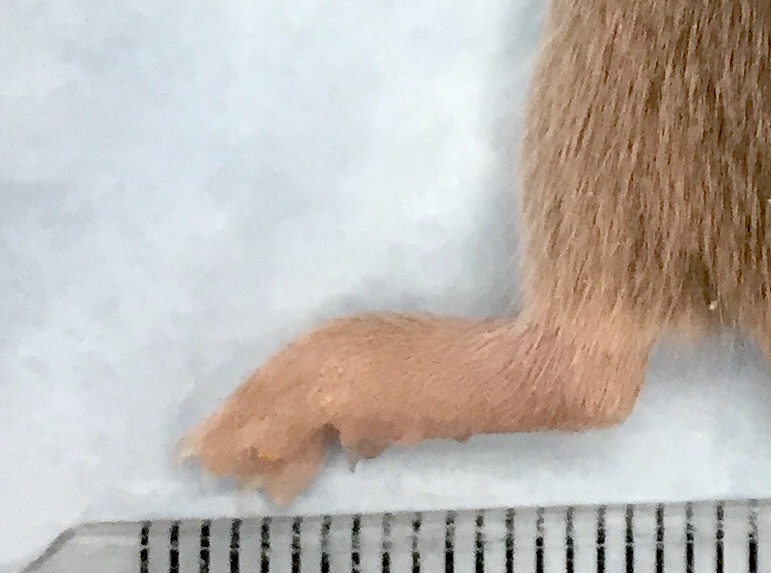

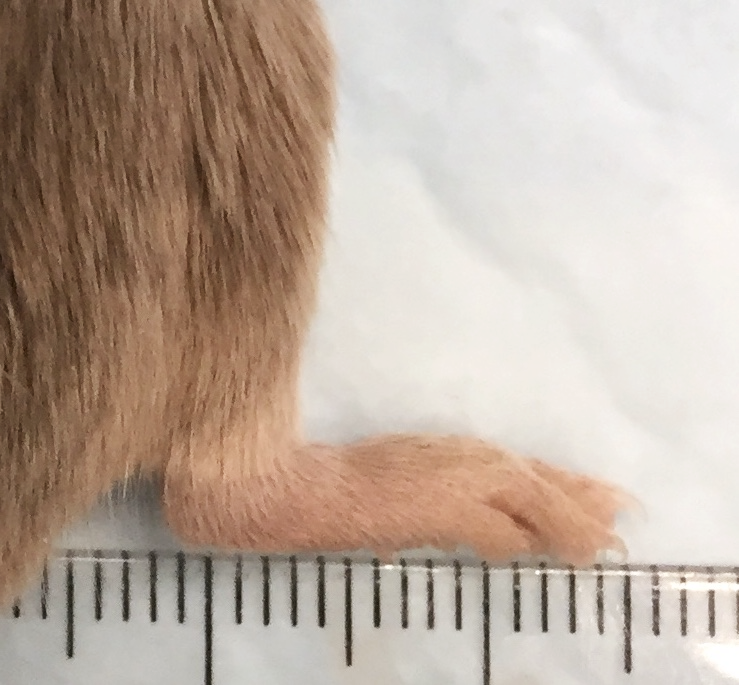


**Figure S1.** Example of Arthritic Scoring. All animals were scored according to instructions provided by Hooke Labs (https://hookelabs.com/protocols/ciaInduction_DBA1.html). **Score 0** = a normal paw; **Score 1 =** one or two toes swollen, with no inflammation of paw or ankle; **Score 2** = Three or more toes inflamed and swollen, but no paw swelling, OR mild swelling of entire paw with no ankle swelling; **Score 3 =** Swelling of entire paw, can include ankle swelling; **Score 4** = Severe swelling of entire paw and all toes, OR ankylosed paw and toes and the mouse cannot grip the wire top of the cage.


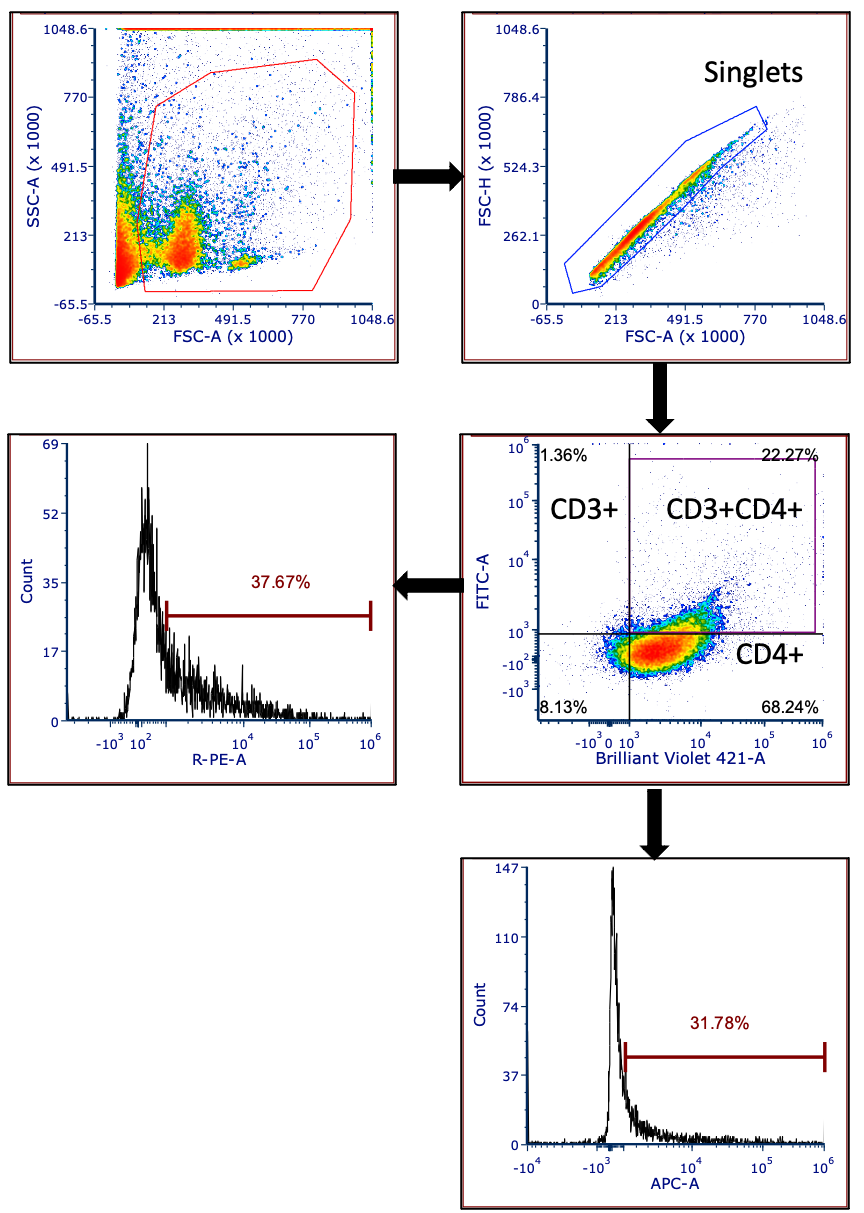


**Figure S2.** Gating strategy for T cell flow cytometry data.


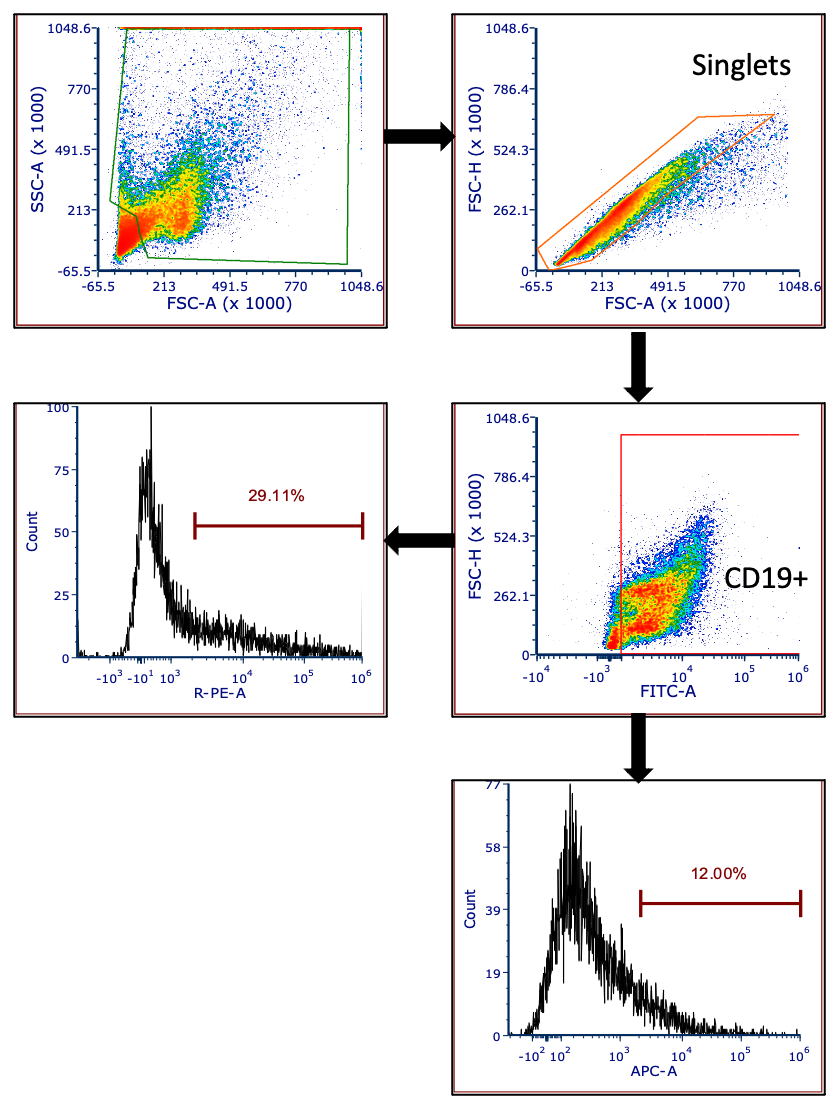


**Figure S3.** Gating strategy for B cell flow cytometry data.
